# Potential Use of Plastic Wastes for Low Thermal Conductivity Concrete

**DOI:** 10.3390/ma11101938

**Published:** 2018-10-11

**Authors:** Artid Poonyakan, Manaskorn Rachakornkij, Methi Wecharatana, Watanachai Smittakorn

**Affiliations:** 1 International Programs in Hazardous Substance and Environmental Management, Graduate School, Chulalongkorn University, Bangkok 10330, Thailand; atidkung@hotmail.com; 2 Center of Excellence on Hazardous Substance Management (HSM), Chulalongkorn University, Bangkok 10330, Thailand; 3 Department of Environmental Engineering, Faculty of Engineering, Chulalongkorn University, Bangkok 10330, Thailand; 4 Civil and Environmental Engineering Department, New Jersey Institute of Technology, Newark, NJ 07102, USA; methi.wecharatana@njit.edu; 5 Department of Civil Engineering, Faculty of Engineering, Chulalongkorn University, Bangkok 10330, Thailand; fcewsk@eng.chula.ac.th

**Keywords:** plastic waste utilization, low thermal conductivity concrete, building material

## Abstract

The use of plastics has increased over the years, thus resulting in a large volume of plastic waste being generated and accumulated in the environment. Due to its non-biodegradability and persistence, recycling processes have become one of the sustainable solutions for preventing environmental deterioration. Plastic wastes, including high density polyethylene (HDPE), low density polyethylene (LDPE), polypropylene (PP), and polyethylene terephthalate (PET), were collected from industrial sector and used as additional ingredients to improve concrete properties. Prior to concrete processing, an increase in wettability of plastic fibers using nonionic surfactant, Dehydol LS-12, was investigated. At the optimal concentration of 10 times of the critical micelle concentration (CMC), an interfacial tension and a contact angle were reduced to 31–32 mN/m and 65°–68°, respectively. Properties of concrete were determined and compared to those of the mortar samples. Porosity was found to increase with higher volume fraction of plastic fibers, whereas decreases in workability, bulk density, thermal conductivity, splitting tensile strength, and compressive strength were encountered. The lowest thermal conductivity was recorded for concrete samples prepared with 30% by volume of LDPE fibers, and the rest in descending order were HDPE, PP, and PET, respectively. Furthermore, the maximal inclusions of plastic fibers were 5% for HDPE and LDPE, 10% for PP, and 50% for PET so as to satisfy the precast concrete wall requirements.

## 1. Introduction

At present, plastic is considered as one of the most important materials of several industries including automobile, electronic appliance, construction, and packaging manufacturers. The rate of plastic consumption has dramatically increased from 200 million tons a year in 2002 to 322 million tons in 2015, and the number is expected to reach 485 million tons in 2030 [1]. Plastic waste is also generated as a non-biodegradable waste, which can cause environmental pollution from unsanitary disposal and toxic leachates and gases, especially carbon monoxide and black smoke produced from open burning [2]. Besides, low bulk density of plastic wastes requires high storage area, thus filling up spaces of sanitary landfills and incapacitating them. Some plastic wastes were dumped into the shoreline or ocean up to 12.7 million metric tonnes in 2010 [3,4] and consequently affected the marine organisms [5]. According to the previous study [5], nano-plastics produced by plastic waste degradation were transferred along the food chain from algae to zooplankton and fish, respectively. 

Utilization of plastic wastes, or plastic recycling, has become a viable and sustainable solution to avoid environmental impacts caused by plastic wastes. Plastic wastes were utilized through recycling and energy recovery (around 48–69%) in 2006 to 2014 [1]. However, plastic waste recycling rate is relatively low in developing countries. This is due to an improper waste management, particularly poor collection and segregation of plastic wastes [6]. Generally, plastic recycling process includes sorting into types based on different polymers, cleaning, chipping, and melting down into pellets. After that, the pellets are used to make plastic products such as plastic bags, containers, carpets, sleeping bags, and jacket insulation materials. The weak points of traditional recycling process are: (1) Cross-contamination among different plastic types and (2) high energy consumption, especially in the melting process. As a result, simple recycling process requiring only cleaning and cutting should be more attractive for waste recycling company and the consumers.

Beneficial properties of recycled plastic are light weight, high chemical resistance, weather durability, and high thermal insulation [7], therefore plastic may have a potential to be recycled as a raw material for construction applications and thermal insulation products. Although some applications of recycled plastic including shotcrete (or Gunite^®^) in tunnels [8] and concrete footpaths [9] have been reported, other applications (i.e., precast concrete wall) should also be considered and possibly applied to green buildings. In Thailand, green building project is promoted to encourage environmentally friendly materials, develop alternative renewable materials, reduce energy consumption, and reduce emission of greenhouse gases (GHGs) [6].

Concrete is widely used that contains basic ingredients such as sand, cement, and water. It is more economical, stronger, and more durable in comparison with wood and asphalt. However, tensile strength of concrete is significantly lower [10,11,12], resulting in easy failure from tensile stresses. Currently, low tensile strength of concrete can be improved by reinforcing it with fibers, especially synthetic fibers [11,13,14,15,16]. There were several studies which attempted to improve concrete properties by recycled waste such as fly ash [17], used tire [18], plastic waste [19,20], PET bottle scrap [12,21,22,23,24,25], recycled PET and PP [7], and melamine waste [26]. This study aims to use macro synthetic fiber made from industrial wastes to reinforce concrete in order to improve its properties. Compressive strengths of plastic materials are typically lower than that of ordinary concrete. When plastic materials are mixed with concrete, they could reduce strength of new composite materials due to the low strength of plastic and poor binding between plastic fibers and concrete microstructures [20,21,27,28]. Hydrophobicity of plastic contributes to poor binding. Nonionic surfactant is then used to increase the wettability of plastic materials which should help to improve the adhesive strength between the plastic materials and concrete.

Concrete is used in either load-bearing structures to bear the main building load (e.g., pile, beam, and foundation) or non-load bearing structures (e.g., concrete wall, insulation, and decoration). Since supporting structures do not require high-strength concrete like those of main structures, plastic fibers concrete will be possible to apply, especially in wall panels. Moreover, thermal conductivity of plastics, especially high density polyethylene (HDPE), low density polyethylene (LDPE), polypropylene (PP), and polyethylene terephthalate (PET), was found to be lower than that of plain concrete [29,30,31]. Generally, concrete walls are not only for dividing space into rooms, but they also help to prevent heat transfer from the elements. In addition, low thermal conductivity concrete becomes an alternative building material for green construction, which is processed by increasing air voids and including high thermal insulation materials into the concrete [32].

To ensure the environmental sustainability and improvement of concrete property, use of plastic waste as reinforcing fibers in the concrete processing is desirable. The objectives of this study are to investigate the possibility of using plastic wastes in construction products such as precast concrete wall. Furthermore, the properties of produced concrete including bulk density, permeable void, thermal conductivity, splitting tensile strength, and compressive strength were also determined.

## 2. Materials and Methods

### 2.1. Plastic Fiber Preparation

Four types of plastic wastes, including high density polyethylene (HDPE), low density polyethylene (LDPE), and polypropylene (PP) collected from a plastic bag manufacturer (T.VIJITPLASTICS, Bangkok, Thailand), as well as polyethylene terephthalate (PET) collected from a PET sheet manufacturer (ROYCE UNIVERSAL Co., Ltd., Nakornpathom, Thailand), were utilized as part of ingredients for concrete production in this study. The plastic wastes were cleaned and cut into 3 mm × 50 mm plastic fibers. Thicknesses of the plastic fibers were approximately 0.05 mm for HDPE and LDPE, 0.1 mm for PP and 0.2 mm for PET, respectively. The four plastic fibers were fit as macro synthetic fibers in which their lengths were 12–65 mm [33] and equivalent diameters (thickness) were <0.3 mm [34]. The average bulk densities of plastic fibers was measured [35]; the values were 142 ± 12 kg/m^3^ for HDPE, 221 ± 22 kg/m^3^ for LDPE, 179 ± 12 kg/m^3^ for PP, and 242 ± 5 kg/m^3^ for PET. The amount of plastic fiber used in the concrete production was calculated according to the different cement volume proportions (5%, 10%, 15%, 20%, 30%, 40%, and 50%).

### 2.2. Cement and Sand Preparation

Portland cement type I and river sand no. 4 (passing through a 4.75 mm sieve) were used as the two main ingredients in the concrete processing. Portland cement mainly composed of Tricalcium silicate (C_3_S), Dicalcium silicate (C_2_S), Tricalcium aluminate (C_3_A), and Tetracalcium aluminoferite (C_4_AF), as well as the trace compounds such as gypsum, free lime, magnesium oxide, and alkaline oxide [36]. The bulk densities of cement and sand were 1506 kg/m^3^ [37] and 1561 ± 12 kg/m^3^ [35] respectively. In addition, the fineness modulus (F.M.) of sand was calculated and its value was approximately 2.75 ± 0.1, which was satisfied for the concrete mixing for fine particles [38,39].

### 2.3. Experimental Conditions and Concrete Processing

In this study, concrete samples were produced using plastic fibers as an additional ingredient under various volume fractions (referring to a ratio of plastic fibers and cement). The ratio of cement to sand was controlled, whereas the water was varied from 15 vol % fraction in order to prevent segregation of reinforced concrete and the plastic fibers were varied from 5% volume fraction to the maximum of 30% volume fraction, as summarized in Table 1. In addition, a nonionic surfactant, Dehydol LS-12 (LS-12) (Thai Ethoxylate Co., Ltd., Rayong, Thailand), was added to increase water adsorption on plastic surface during the concrete processing [40]. Brief procedure for concrete processing was as follows; (1) dissolve the nonionic surfactant in the water, and then soak the plastic fibers in the solution, (2) add the sand in a concrete mixer and operate it for one min, (3) add the soaked plastic fibers in the mixer, and then mix for 1–2 min, (4) add cement and mix for 1–2 min, (5) add the nonionic surfactant solution and mix for 2 min, and (6) test the produced concrete in accordance with slump test [41], making and curing of cylinder concrete [42], bulk density and porosity [43], thermal conductivity [44], splitting tensile strength [45], compressive strength test [46] (ASTM C143, C192, C642, C518, C496, and C39) standards. Further, the properties of the produced concrete were compared to the properties of control mortar samples, which contained no plastic fibers.

### 2.4. Plastic Fiber Test

**Figure 1:**
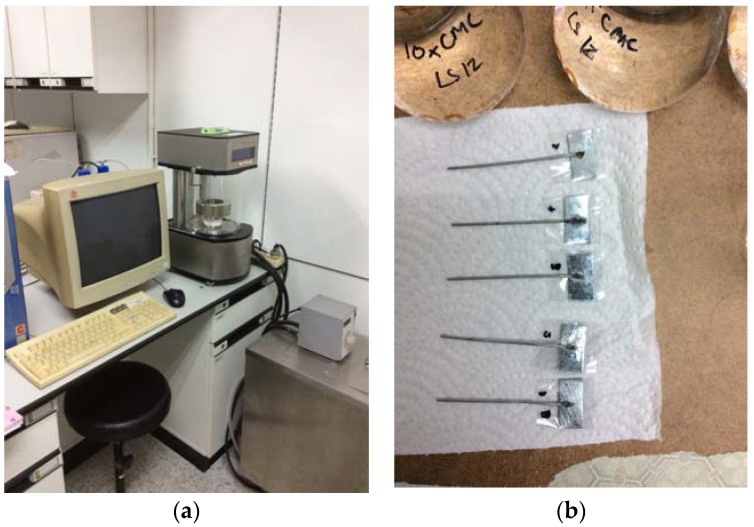
(**a**) Surface tension and contact angle measurement using a DCAT 11 with cooler, and (**b**) Plastic samples in a 1 cm × 2 cm size.

Prior to the concrete processing, wettabilities of plastic fibers were determined to optimize the nonionic surfactant amount. Two parameters; namely, surface tension (γSL) and contact angle (θc) were used to measure the wettability. Brief procedure includes preparing the plastic fibers of HDPE, LDPE, PP, and PET in a 1 cm × 2 cm size, and measuring the surface tension and contact angle at various LS-12 solution concentrations from 1 to 70 times of its critical micelle concentration (CMC) at 25 °C by DCAT 11 (IP-HSM laboratory at Chulalongkorn University, Bangkok, Thailand) or Dynamic contact angle and tension meter as shown in Figure 1.

### 2.5. Concrete Test

(1) Workability

The fresh concrete from plastic waste was measured for workability by slump test [41]. The testing procedure was as follows; (1) pour the fresh concrete in the slump mold for three layers (each layer was tamped in 25 strokes), (2) cut the top by trowel when the slump mold was full, (3) pull out the slump mold vertically and put aside the produced concrete, and (4) measure the subsidence value. 

(2) Bulk density and porosity 

The hardened concrete was evaluated for the bulk density and porosity. The testing procedure was as follows; (1) measure the dry weight of produced concrete after heating at 100–110 °C in an oven and cooling down to 20–25 °C in a desiccator, (2) immerse the concrete in the water at 21 °C for 48 h, and then measure the weight, (3) boil the concrete for five hours, and then measure the weight after cooling down, and (4) suspend the concrete in the water, and measure the immersed apparent mass [43].

(3) Thermal conductivity

The cube concrete with a 30 cm × 30 cm × 7.5 cm size was prepared, and the heat flux was measured using NETZSCH HFM 436 equipment [44] (Department of Science Service, Bangkok, Thailand). Furthermore, the thermal conductivity (k) and thermal resistance (R) were calculated via the following Fourier’s law as shown in Equations (1) and (2).

(1) $$Q=-k\frac{\mathrm{dT}}{\mathrm{dx}}$$

where Q is heat flux (W/m^2^), k is thermal conductivity (W/m·K), dT is temperature difference between both sides (K or °C), and dx = thickness (m)

(2) $$R=\frac{\mathrm{dx}}{k}$$

where R is thermal resistance (m^2^·K/W).

(4) Splitting tensile strength

The splitting tensile strength was examined according to American Society for Testing and Materials (ASTM) C496 [45] by molding four replicates of concrete cylinder with a 15 cm diameter and 30 cm length for 24 h. The concrete samples were cured in a lime-saturated pond for 28 days, and measured their dimensions and weights [42] and finally splitting tensile strength.

(5) Compressive strength

The compressive strength was evaluated according to ASTM C34 [46] by molding four replicates of concrete cylinders. The concrete samples were cured using the same method of splitting tensile strength test. The compressive strength values of produced concrete were compared with Thailand’s precast concrete wall panel standard (TIS 2226-2548) [18,46].

All parameters above are explained with mean and standard deviation (SD) values.

## 3. Results and Discussions

### 3.1. Property of Plastic Fibers

**Figure 2:**
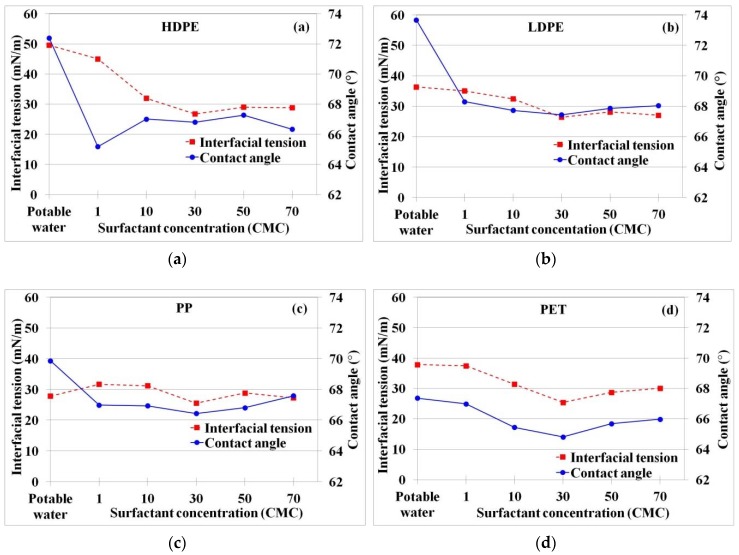
Change of interfacial tension and contact angle at various surfactant concentrations; (**a**) high density polyethylene (HDPE), (**b**) low density polyethylene (LDPE), (**c**) polypropylene (PP), and (**d**) polyethylene terephthalate (PET) fibers.

Prior to concrete processing, the wettabilities of plastic fibers were determined under various nonionic surfactant concentrations (as presented in CMC) using interfacial tension (γSL) and contact angle (θc). From Figure 2, the average interfacial tension between HDPE fiber and water was 50 ± 8 mN/m, which was the highest value among the four fibers. The interfacial tension decreased to the average minimal value of 27 ± 0 mN/m when surfactant concentration increased from 1 to 30 CMC. On the other hand, the interfacial tension showed a slight increase at the high surfactant concentration of >30 CMC. The results showed that surfactant at optimal concentration could aid in increasing wettability on plastic surface. In the meantime, the average contact angle of HDPE sharply decreased from 72° ± 2° to 65° ± 4°–67° ± 1°, when the nonionic surfactant was used (Figure 2). The results demonstrated that the addition of nonionic surfactant improved wettability of HDPE fiber. The similar change of interfacial tension and contact angle was observed for LDPE, PP, and PET fibers, however the improvement on plastic fibers wettability was smaller than that of the HDPE fiber (Figure 2b–d)). Taking into account cost-effectiveness, optimal surfactant concentration was found at 10 CMC. This value was used as a controlled parameter throughout the experiments.

### 3.2. Property of Concrete

#### 3.2.1. Workability

**Figure 3:**
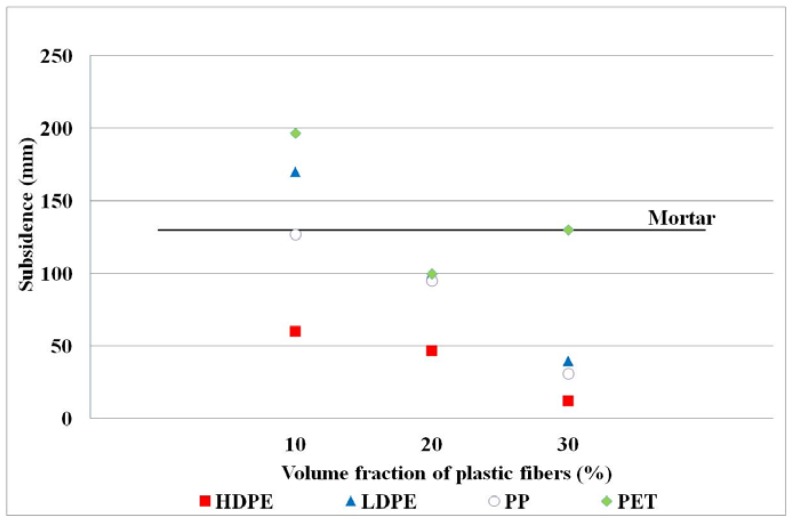
Workability of fresh concrete. The subsidence of mortar (control sample) is in the solid line.

**Figure 4:**
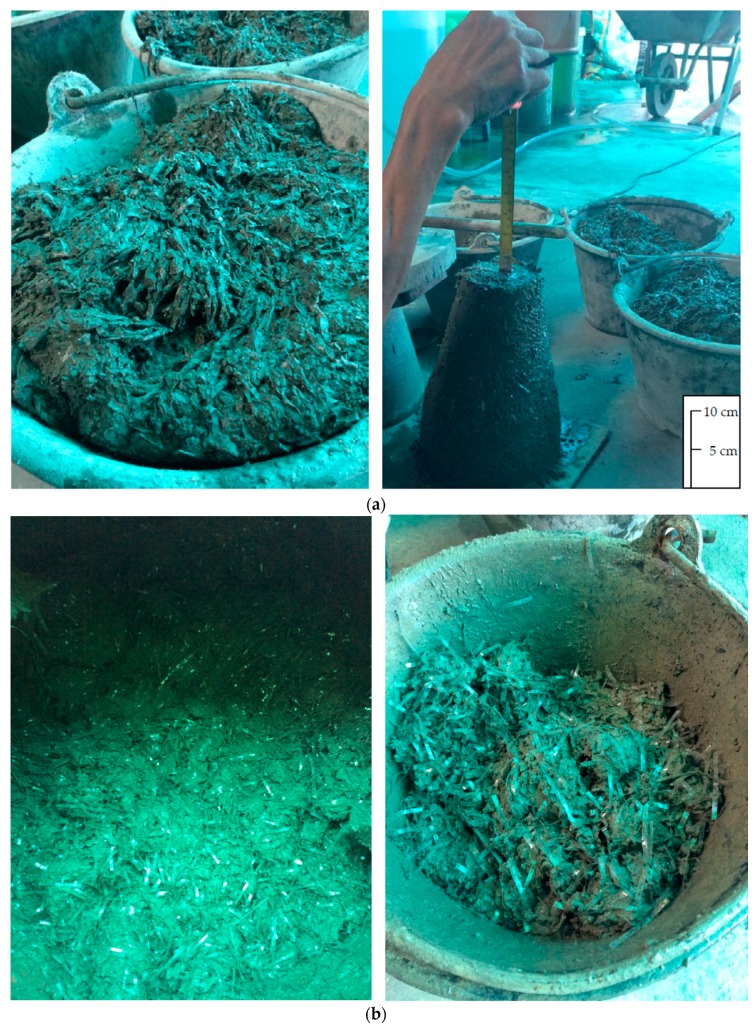

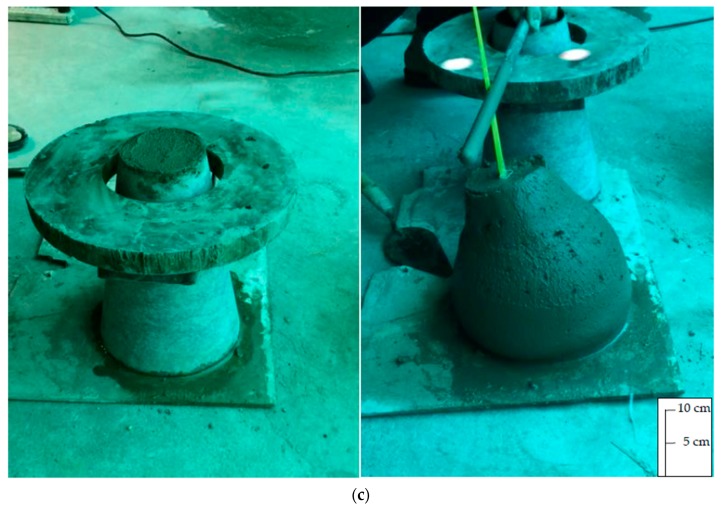
Photographs of workability test during concrete processing: (**a**) Complex network of 20 vol % HDPE fibers in concrete, (**b**) Segregation of 30 vol % PP fibers in concrete, and (**c**) Mortar (control sample without plastic fibers).

Workability of fresh concrete using HDPE, LDPE, PP, and PET fibers was represented by subsidence values, and the results were shown in Figure 3. The subsidence values were found to have decreased with increasing volume fraction of plastic fibers [13,47,48]. Movement of fresh concrete/mortar, which is primarily the cause of workability or slump, is restricted by the presence of recycled plastic fibers. The occurrence of complex network structure during the concrete processing is presented in Figure 4a. In addition, increasing the surface area of plastic fibers at high volume fractions can improve the cement paste absorption and the viscosity of fiber reinforced concrete [14,20,48,49]. The low subsidence value refers to the low workability of fresh concrete, which can cause segregation in building structures. Fresh concrete dissociation brings about fracture on hardened concrete, and consequently losing concrete strength. To prevent segregation of plastic fibers (see Figure 4b) in the reinforced concrete composites, two recommendations were proposed [13,47,48]: (1) Increasing the amount of water used in the mix and (2) adding water reducing agents. Increasing water to cement (w/c) ratio was appropriate for this study in order not to cause chemical interaction with the non-ionic surfactant. That w/c ratio was gradually increased from 15% volume fraction so as to help reduce honeycomb concrete [50]. From Figure 3, the HDPE and PP fibers at various volume fractions provided lowered subsidence values in comparison with that of the mortar (Figure 4c). In the meantime, the LDPE and PET fibers offered lower subsidence values than that of the mortar, when the volume fraction was 20% and 30%. Relatively low subsidence values of 1–6 cm were obtained when the HDPE fiber was utilized in the concrete. According to these results, PET was observed to be more rigid than other plastics, so addition of PET fibers to the concrete was likely to agglomerate and form fiber balls [51]. PET fiber balls (even though they are hydrophobic) can adsorb water within voids during concrete processing. When produced concrete was put into the slump cone and tamped, water retained in PET balls could be released. Finally, increasing the amount of water in the mixture would increase subsidence value.

#### 3.2.2. Bulk Density and Porosity

**Figure 5:**
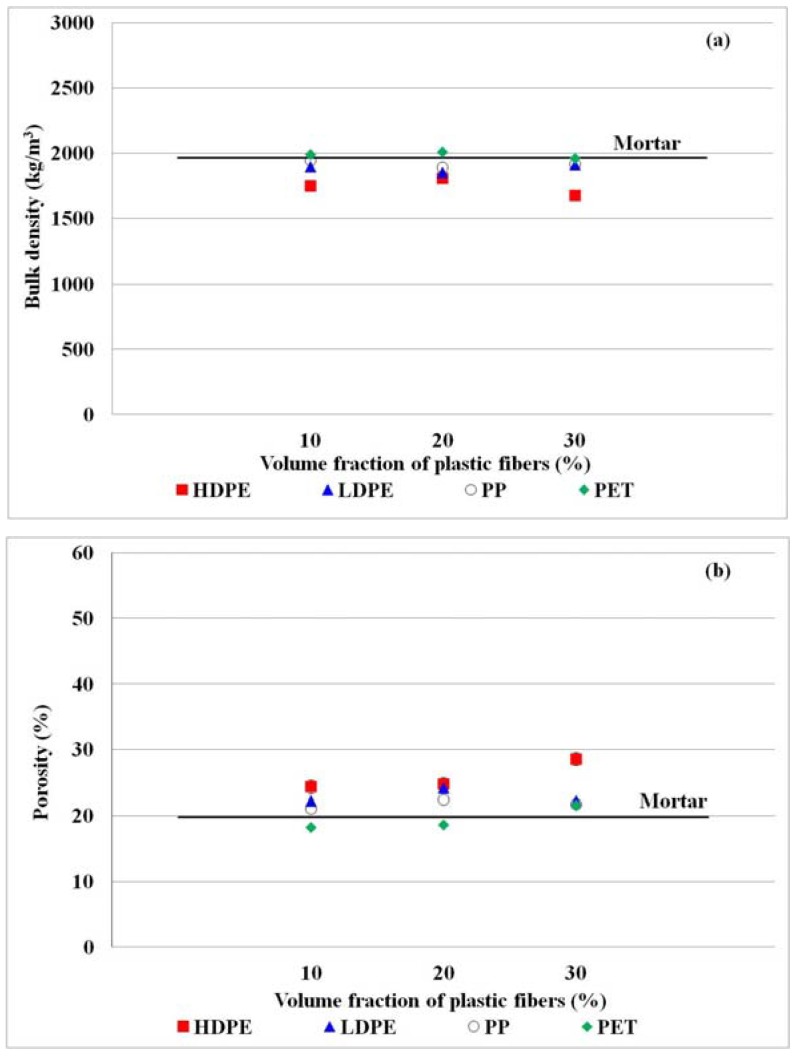
Relationship between volume fraction of plastic fibers and (**a**) bulk density and (**b**) porosity of produced concrete. The bulk density and porosity of mortar (control sample) are in the solid line.

In this study, bulk density and porosity of the produced concrete were also affected by the increase in volume fraction of plastic fibers. The use of plastic fibers as the additional ingredient resulted in lower bulk density of produced concrete than that of the mortar [21,27,52]. This is because the complex network structure from plastic fibers resulted in honeycombs [16,50,53]. The lowest bulk density of approximately 1677 kg/m^3^ on average was obtained in the produced concrete from HDPE fiber, and the bulk densities of the produced concrete with LDPE, PP and PET were about 1853, 1892, and 1960 kg/m^3^ on average, respectively (Figure 5a). This is due to the fact that the surface tension between plastic and water of HDPE was found to be higher than other plastics, and consequently inducing air void generation. The permeable voids (porosity) were negatively correlated to the bulk density so the lower bulk density of produced concrete than the mortar was observed, especially the produced concrete from HDPE fiber (Figure 5b). Therefore, the produced concrete in this study can be further developed to be lightweight concrete with high insulation.

#### 3.2.3. Thermal Conductivity

Thermal conductivity is inversely proportional to thermal resistance. Thermal conductivity depends on the material property, surface area, thickness, and temperature gradient in steady state heat transfer condition [18]. According to Fourier’s law [54] Equation (1), thermal conductivity is the term for material characteristic which can transfer heat from higher to lower temperature sides. According to Klein [29], thermal conductivities of HDPE, LDPE, and PP were found to be about 0.43, 0.35, and 0.23 W/(m·K) at 25 °C, while thermal conductivities of ordinary concretes with various aggregates were normally about 1.34–2.92 W/(m·K), three to thirteen times higher [30]. Similarly, Tae Sup Yun et al [31] found that the thermal conductivities of mortar and concrete were about 2.0 W/(m·K). Plastic fibers are expected to help reducing thermal conductivities of concrete due to its property. 

**Figure 6:**
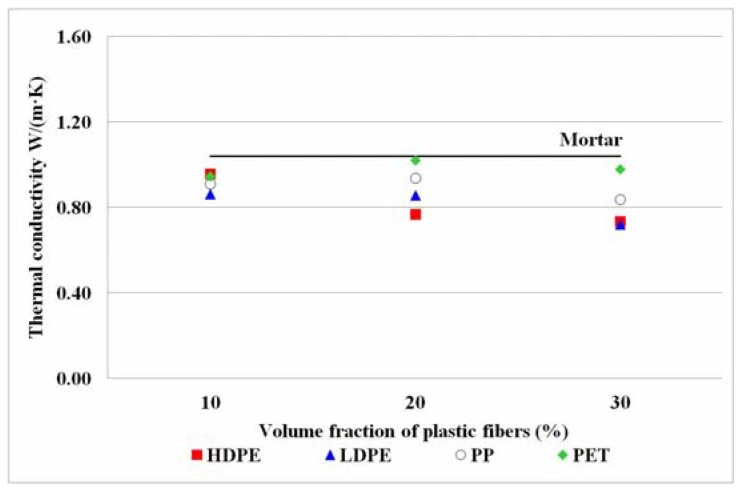
Thermal conductivity of produced concrete. The thermal conductivity of mortar (control sample) is in the solid line.

The thermal conductivity (k) of the produced concrete was calculated from the experiments, and the results were shown in Figure 6. The thermal conductivities of concrete mixed with recycled HDPE, LDPE, PP, and PET were about 0.74–0.96, 0.72–0.86, 0.84–0.94, 0.95–1.02 W/(m·K) on average at 25 °C, respectively, whereas the thermal resistances of fiber reinforced concrete in Equation (2) were found to be about 0.08–0.11, 0.09–0.11, 0.08–0.09, and 0.07–0.08 m^2^·K/W, respectively. Apparently, the thermal conductivity values of produced concrete from all plastic fibers decreased with increasing volume fractions of plastic fibers, and their thermal conductivities were lower than that of the mortar by about 2–31%. This is concordant with the work of Fraternali et al [7] who studied recycled PET fiber and virgin PP at 1% in concrete and the thermal conductivities were found to have reduced when comparing to the thermal conductivities in plain concrete. Not only is synthetic fiber property affecting the thermal conductivity of fiber-reinforced concrete but small permeable void [32,55] can also restrain heat transfer as well. According to Figure 5, the more volume fractions of synthetic fibers are mixed in concrete, the more permeable voids of fiber-reinforced concrete were found [16,56]. The results demonstrated that the plastic fibers of HDPE, LDPE, PP, and PET can decrease the thermal inducing property or heat transfer of produced concrete. The lowest thermal conductivity of 0.7–0.9 W/(m·K) was found in the produced concrete from LDPE fiber, which had higher porosity than the control. The thermal property improvement of produced concrete was in the following order; LDPE > HDPE > PP > PET. Furthermore, the thermal resistance values (R) of produced concrete were calculated, the maximal value of 0.1 m^2^·K/W was recorded in the produced concrete at 30% volume fraction of LDPE fiber, which was verified to be a good thermal insulator. Therefore, the produced concrete from LDPE fiber has an energy-saving potential when it is applied as a green building material. In fact, one of the green building requirements promoted in Thailand is low energy consumption, therefore if this composite material is used in concrete precast wall or non-load-bearing structure, it can insulate heat transfer from side to side better than the ordinary mortar and help to reduce energy consumption from room temperature adjustment.

#### 3.2.4. Tensile and Compressive Strengths

(1) Splitting Tensile Strength

Normally, tensile strength of concrete is low when compared with its compressive strength, as a result, high strength materials, such as, steel, plastic, etc., are introduced into the mixture to improve the brittle property of the cementitious composites. According to Hasan et al. [13], adding 0.33–0.51% by volume of macro PP synthetic fibers in concrete could enhance about 10–15% of the splitting tensile strength. Other investigators had also found similar results [11,14,15,16]. Choi and Yuan [11] reported that adding 1–1.5% by volume of PP fibers in concrete increased the splitting tensile strength of concrete by 50%, and Hsie, Tu, and Song [14] increased the splitting tensile strength (9–13%) of concrete by mixing 3.6–9.6 kg/m^3^ of PP hybrid fibers with concrete.

**Figure 7:**
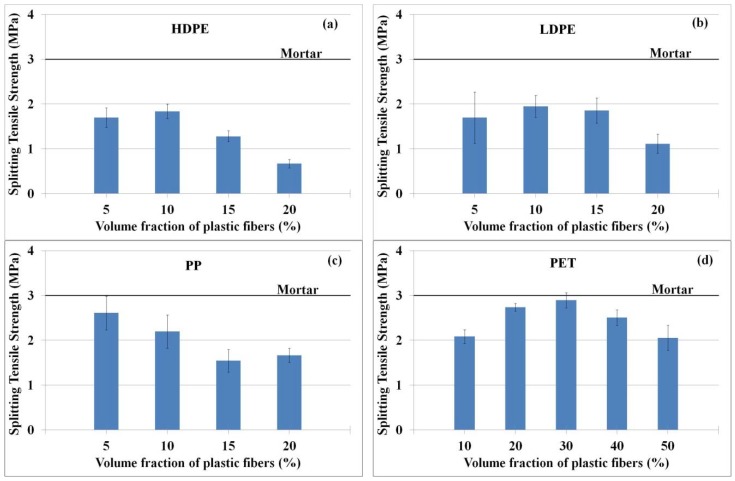
Splitting tensile strengths of produced concrete; (**a**) HDPE, (**b**) LDPE, (**c**) PP, and (**d**) PET fibers. The splitting tensile strength of mortar (control sample) is in the solid line.

Ductile fibers, when added into cementitious composites, can enhance its tensile strength depending on several factors such as fiber toughness, fiber volume fraction, alignment, and bonding between the fibers and the cementitious matrix. In this study, the optimum volume fractions of recycled HDPE, LDPE, and PET plastic to achieve the highest splitting tensile strength of the fiber-reinforced concrete composites were found to be 10%, 10%, and 30%, respectively. Lower and higher volume fraction than these optimum amounts resulted a lower splitting tensile strength. For the case of PP recycled plastic, optimum splitting tensile strength of the composites was found to be at 5% volume fraction, thereafter, at higher volume fractions the tensile strengths of the composites reduced (see Figure 7). In general, all the mixings in this study with different fiber volume fractions showed lower splitting tensile strengths than that of plain mortar (about 3 MPa). The strength reduction varied from 4 to 80% depending on type of fiber and volume fraction. Similar findings were reported by several investigators [11,16,21,27,50,52] that increasing fiber volume fraction could cause weak bonding between plastic fiber and cementitious matrix, thus reducing its effectiveness in strengthening the concrete composites. Besides, the splitting tensile strength reduction (when mixed with 25–75% fiber volume fraction) was reported to be lower by 41% when compared with the tensile strength of normal concrete [11]. As mentioned earlier, the lower strength might be attributed to the high volume fractions of plastic fibers used in this study. Larger volume fraction often leads to clumping or balling of fibers [51], making them less effective in strengthening the concrete composites. It should be noted that one of the objectives of this study is to explore the potential utilization of recycled plastics in concrete as alternative to typical disposal in landfill. The use of large quantity of recycled plastic fibers is therefore a goal of this investigation, if the final fiber-reinforced cementitious composites can be used as construction materials, such as wall panel, etc.

(2) Compressive Strength

**Figure 8:**
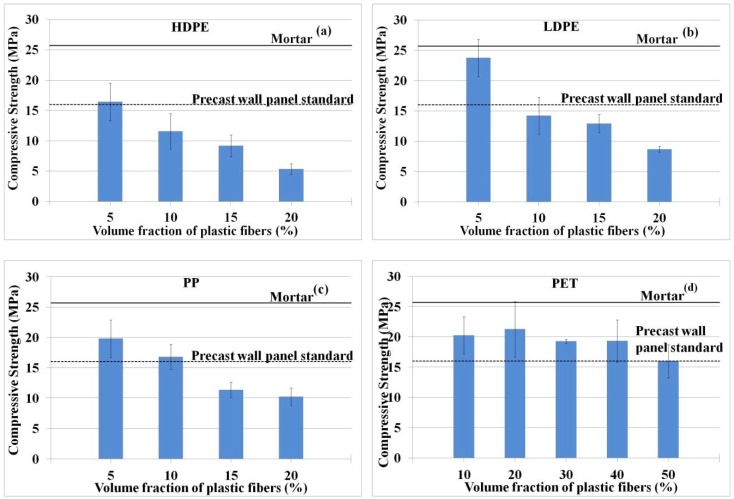
Compressive strengths of produced concrete; (**a**) HDPE, (**b**) LDPE, (**c**) PP, and (**d**) PET fibers. The compressive strength of mortar (control sample) and the standard of compressive strength for precast concrete wall panel are denoted with solid lines and dash lines, respectively.

Concrete is commonly known for its high compressive strength with a rather low tensile strength, typically about one-tenth of its compressive strength. The addition of low fiber volume content (typically less than 1% for plastic fibers such as PP) into concrete often does not affect the compressive strength of concrete [7,11,13,24,57]. In some circumstances, the presence of fibers can enhance some property of concrete composites [7,11,13,14,15,16], for instance, tensile strength, micro crack reduction, crack opening reduction, and so on. However, the large volume fraction of fibers often lowers the compressive strength of concrete as the hydrophobic property of plastics is likely to increase air void [16,50,58] in the cementitious composites. Figure 8 shows the compressive strength of concrete produced with the addition of HDPE, LDPE, PP, and PET recycled plastic fibers. The volume fraction of fibers used in this study varies from 5 to 50%. It was found that the compressive strength values of all the mixings, regardless of the fiber type and fiber volume fraction, were likely to be declined with increase in plastic contents. With a 5% fiber volume fraction, mixing with HDPE, LDPE, and PP recycled fibers showed a strength reduction of −36%, −8%, and −23%, respectively, when compared to mortar. The strength reduction escalated to −79%, −66%, and −60% when fiber volume fraction increased to 20%. However, concrete with PET recycled plastics showed a better performance with lesser strength reduction, ranging from only −38% to −17% when the fiber volume fraction increased from 10% to 50%. Similar results of strength reduction were also reported by other investigators [20,21,28] when percentage of fiber volume fractions used in the concrete was increased.

Typical compressive strength of concrete used in the USA construction industry varies from 17 MPa for residential buildings to 28 MPa for commercial buildings [59]. As stated, one of the objectives of this study was to investigate the potential utilization of recycled plastics of materials for concrete wall panel, which must adhere to the TIS 2226-2548 Standard for precast concrete wall panel. It is required that all concrete used for precast wall panels (non-structural load carrying) must have a minimum compressive strength of 16 MPa [60]. In this study, only concrete mixing with HDPE and LDPE recycled plastics with 5% fiber volume fraction and PP with 10% fiber volume fraction produced sufficient compressive strength to be used for precast wall panel application. As for PET recycled plastic, all mixings (with 10% to 50% fiber volume fraction) tested in this study met the minimum compressive strength requirement for precast wall panel application. Clearly, this study shows that recycled plastics, when used in fiber-reinforced concrete, have a good potential to be used as construction materials for many non-structural load carrying members in the building system, thus paving the way for a new type of environmental friendly renewable construction materials [25,52].

From all experiments and characterization, HDPE, LDPE, and PP have the potential to be used as additional ingredients to hinder concrete’s thermal conductivity and can be used for manufacturing of high insulation material. However, the property was found to be more effective in the produced concrete from LDPE fiber, and to a lesser degree from HDPE and PP fibers. Though PET fiber was found unsuitable for thermal resistance property in concrete because of its inability to show clear improvement over regular mortar, PET fiber compressive strength and splitting tensile strength were higher than other fibers, which was the advantage for utilizing PET waste in green building.

## 4. Conclusions

The industrial plastic wastes (HDPE, LDPE, PP, and PET) were recycled as reinforcing fiber in concrete processing in this study. The addition of nonionic surfactant solution was required to improve the wettability of plastic fiber surface. At the optimal concentration of 10 CMC, the interfacial tension and contact angle were in the range of 31–32 mN/m and 65°–68° on average, respectively. The results showed that the contact angles of both HDPE and LDPE were distinctively reduced by LS-12 solution at 10 CMC. The properties of fresh and hardened concretes containing plastic fibers were determined and compared with those of the controls (mortar) which contained no plastic fiber. Workability values of all plastic fibers applied in concrete were likely to be decreased with increase fibers content due to complex network structure but 30% PET, which was reversed, might be the cause of fiber agglomeration. Besides, adding fiber contents in concrete could increase the porosity and decrease the bulk density because hydrophobic property of plastics was able to generate air void inside concrete. As a result of porous concrete, the results demonstrated that the HDPE, LDPE, and PP fibers could enhance lowering of thermal conductivity. However, the concrete prepared with PET fibers resulted in the similar property to that of the mortar. Moreover, splitting tensile strength and compressive strength of concrete were not improved by 5–50 vol % plastic contents. The best concrete property was observed when the LDPE fiber was utilized at 30% of volume fraction; subsidence was 4.0 cm, bulk density was 1911 kg/m^3^, porosity was 22%, and thermal conductivity was 0.72 W/(m·K) on average. According to the standard of precast concrete wall panel in Thailand, the optimal volume fraction of LDPE fiber should be reduced to 5–10% to maintain the compressive strength of 16 MPa. These alternative renewable materials not only improve lower thermal conductivity (2–31%) of plain concrete but also help solve plastic wastes in our environment. Consequently, they would be recommended for green building in non-load-bearing structure.

More study should be focused on the surfactant role in synthetic fiber reinforced concrete. Moreover, the degradation of plastic fiber in hardened concrete should be considered when applying this composite material as concrete wall panel.

## Figures and Tables

**Table 1:** Conditions of concrete processing from various plastic wastes.

Experimental Conditions	Water-to-Cement (W/C) Ratio	Cement (kg)	Sand (kg)	Water (kg)	Plastic Fiber (kg)
(1) Mortar	0.50	33.00	66.00	16.50	-
(2) 5% volume fraction					
HDPE	0.50	30.12	60.24	15.06	0.14
LDPE	0.50	30.12	60.24	15.06	0.22
PP	0.50	30.12	60.24	15.06	0.18
PET	0.50	30.12	60.24	15.06	0.24
(3) 10% volume fraction					
HDPE	0.50	30.12	60.24	15.06	0.28
LDPE	0.50	30.12	60.24	15.06	0.44
PP	0.50	30.12	60.24	15.06	0.36
PET	0.50	30.12	60.24	15.06	0.48
(4) 15% volume fraction					
HDPE	0.55	30.12	60.24	16.57	0.42
LDPE	0.55	30.12	60.24	16.57	0.66
PP	0.55	30.12	60.24	16.57	0.54
PET	0.55	30.12	60.24	16.57	0.72
(5) 20% volume fraction					
HDPE	0.60	30.12	60.24	18.07	0.56
LDPE	0.55	30.12	60.24	16.57	0.88
PP	0.55	30.12	60.24	16.57	0.72
PET	0.55	30.12	60.24	16.57	0.96
(6) 30% volume fraction					
HDPE	0.60	30.12	60.24	18.07	0.84
LDPE	0.55	30.12	60.24	16.57	1.32
PP	0.55	30.12	60.24	16.57	1.08
PET	0.55	30.12	60.24	16.57	1.44
(7) 40% volume fraction					
HDPE	-	-	-	-	-
LDPE	-	-	-	-	-
PP	-	-	-	-	-
PET	0.55	30.12	60.24	16.57	1.92
(8) 50% volume fraction					
HDPE	-	-	-	-	-
LDPE	-	-	-	-	-
PP	-	-	-	-	-
PET	0.55	30.12	60.24	16.57	2.40
